# Impact of body mass index and metabolically unhealthy status on mortality in the Japanese general population: The JMS cohort study

**DOI:** 10.1371/journal.pone.0224802

**Published:** 2019-11-07

**Authors:** Toshihide Izumida, Yosikazu Nakamura, Shizukiyo Ishikawa

**Affiliations:** 1 Kamitaira Clinic, Nanto, Toyama, Japan; 2 Division of Public Health, Center for Community Medicine, Jichi Medical University, Shimotsuke, Tochigi, Japan; Beijing Key Laboratory of Diabetes Prevention and Research, CHINA

## Abstract

This study aimed to investigate the associations of body mass index (BMI) and metabolically unhealthy weight with all-cause mortality, cardiovascular disease (CVD) mortality, and cancer mortality as well as the effect of age on the associations. This prospective study enrolled Japanese individuals in the general population. Participants were divided into eight phenotypes according to the BMI classification and metabolic status. Hazard ratios (HRs) and 95% confidence intervals (CIs) were calculated using a Cox regression hazard model. In total, 10,824 individuals with a mean age of 55.3 years were evaluated. During a mean follow-up of 18.4 years (198,776 person-years), 2,274 participants died. Among the metabolically unhealthy, the association between BMI and mortality was J-shaped after adjustment for various confounders (multivariable HR [95% CI] for all-cause mortality: underweight, 2.0 [1.5–2.7]; obesity 2.8 [2.1–3.6]). The association remained the same in metabolically unhealthy participants aged <65 years and ≥65 years. The results were compatible in the analyses restricted to subjects who never smoked. Regardless of age, metabolically unhealthy underweight (MUHU) have approximately a 3-fold higher risk of CVD mortality, compared with metabolically healthy normal weight. Not only metabolically unhealthy obesity, but also MUHU was strongly associated with an increased risk of mortality. More attention should be given to the health issues of metabolically unhealthy participants without obesity, particularly those with MUHU.

## Introduction

Obesity is often accompanied by metabolic syndrome (MetS) due to excess visceral adipose tissue and is an important public health and socioeconomic problem worldwide [1,2,3,4,5]. Both obesity and MetS are associated with increased morbidity and mortality from cardiovascular disease (CVD) and cancers [3,6,7,8,9]. Since 2008, nationwide lifestyle interventions have been provided for individuals identified to have abdominal obesity, high body mass index (BMI), and metabolic risk factors in Japan. The program has effectively improved abdominal obesity and metabolic dysfunctions, such as raised blood pressure, impaired glucose tolerance, and dyslipidemia [10].

However, some normal weight or underweight individuals might have also developed unexpected metabolic dysfunction with insulin resistance induced by several different pathways that deviate from the dose-response relationship between body fat burden and metabolic disturbances [8,11,12]. These individuals have been described as “metabolically unhealthy normal weight” (MUHNW) and “metabolically unhealthy underweight (MUHU).” Several epidemiologic studies showed that in Asian populations, such as the Chinese, normal weight and underweight individuals have comparable metabolic disorders with the obesity [8,13]. Therefore, more attention might be needed for the health issues of the MUHNW and MUHU [3,8,14]. MUHNW has a greater than 1.3- to 3-fold higher risk of cardiovascular events or all-cause mortality than metabolically healthy normal weight (MHNW) [9,12,15]. *Bea et al*. suggested that MUHU tended to be at increased risk of CVD events or mortality (3); however, the data have been derived from Western populations, and it is unclear whether these also hold true for Asian populations because Asians have higher body fat percentages, greater abdominal and visceral fat deposition, and less muscle mass and connective tissue than Western at the same body mass index (BMI) [16,17]. Asians, such as the Japanese, also have lower insulin resistance and insulin secretion than Westerners at the same level of visceral adipose tissue [14], and the mechanisms involved in the development of insulin resistance and metabolic dysfunction might differ between Asian and Western populations. Accordingly, evidence about the impact of BMI and metabolically unhealthy on mortality, particularly in Asians, is limited, and a prospective study is required to determine whether metabolically unhealthy subjects without obesity also have a high risk of all-cause, CVD, or cancer mortality. Several previous studies showed the strong effects of age on the association between BMI, or MetS, and mortality [16,18,19].

Therefore, this study aimed to investigate the associations of BMI and metabolically unhealthy with mortality. Towards this goal, we conducted a prospective study in a Japanese general population.

## Materials and methods

### Population

The present study was conducted as part of the Jichi Medical School (JMS) Cohort Study, which is a prospective, population-based study of 12,490 participants aimed to evaluate the relationship between the risk factors of atherosclerosis and CVD in the Japanese general population. Details of the JMS Cohort Study design and some descriptive data are available in a previous report [20].

This study evaluated Japanese individuals who had underwent basic health screening that was conducted in accordance with the medical care system for the elderly. The study covered 12 rural districts in Japan, and baseline data were collected between April 1992 and July 1995. In each community, a local government office sent personal invitations for mass CVD screenings to all the subjects by mail. All the participants provided written informed consent prior to inclusion. Among them, complete follow-up was achieved for 99% (n = 12,388) of the cohort. Individuals with insufficient data in at least one of the following parameters were excluded: body mass index (BMI) (n = 504); blood pressure (BP) (n = 442); high-density lipoprotein cholesterol (HDL-C) (n = 155); triglyceride (TG) (n = 157); blood glucose (n = 174); total cholesterol (n = 156); smoking status (n = 925); or medical history of hypertension (n = 1,059), dyslipidemia (n = 1,145), diabetes mellitus (n = 1,139), myocardial infarction (n = 1,158), stroke (n = 1,152), and cancer (n = 1,165). This study was approved by the Institutional Review Board of Jichi Medical School (Tochigi, Japan) (Epidemiology 06–11).

### Measurements

Previous reports have described the baseline examination in detail [20,21]. Briefly, the participants’ height and weight were measured using a standardized method, with 0.1 cm and 0.1 kg units of measurement, respectively. Height was measured with stockinged feet. Weight was recorded with the subjects clothed. BMI was calculated as weight in kilograms divided by height in meters squared (kg/m^2^). Systolic and diastolic BPs were measured via a fully automated sphygmomanometer (BP203RV-II; Nippon Colin, Komaki, Japan) placed on the right arm of subjects, who had been resting while seated for 5 minutes before measurement.

Blood samples were collected to measure total cholesterol, HDL-C, TG, and blood glucose. Total cholesterol and TG were measured using an enzymatic method (Wako, Osaka, Japan; inter-assay coefficient of variation (CV): 1.5% for total cholesterol and 1.7% for TG). HDL-C was measured via the phosphotungstate precipitation method (Wako, Osaka, Japan; inter-assay CV: 1.9%). Blood glucose was measured via an enzymatic method (Kanto chemistry, Tokyo, Japan; inter-assay CV: 1.9%).

Information about medical history and lifestyle was obtained using a self-report questionnaire. Past and present illness history was assessed, and medication information for present illness was obtained. Smoking status was classified as never, ex-, or current smoker. Physical activity was assessed according to the criteria in The Framingham Study. Data from the physical activity questionnaire was used to determine the physical activity index (PAI), which was calculated by summing the products of the hours spent at 5 activity levels and weighted factor based on the oxygen consumption required for the activity: 1.0 for sedation including sleeping; 1.1 for quiet working, such as sitting; 1.5 for a light level of working such as standing; 2.5 for a moderate level of working; and 5.0 for heavy work, during a typical workday [22].

### Definition of underweight, normal weight, pre-obesity, obesity, and metabolically unhealthy profile

The participants were classified into four groups according to their BMI using cut-offs proposed by the World Health Organization [23]: underweight, <18.5 kg/m^2^; normal weight, 18.5–24.9 kg/m^2^; pre-obesity, 25.0–29.9 kg/m^2^; and obesity, ≥30.0 kg/m^2^. MetS was defined according to the modified Adult Treatment Panel III criteria [24].

The waist circumference was excluded as a criterion because our main purpose was to evaluate the prognosis of the BMI-stratified metabolically unhealthy participants regardless of their body fat distribution and visceral adipose tissue. In a few studies, the presence of insulin resistance and systemic low-grade inflammation was supposed to represent metabolically unhealthy [25,26]. However, most epidemiologic and clinical studies did not determine both insulin resistance and inflammation, and the subjects were considered metabolically unhealthy when no less than 2 components of MetS were present [1,2,8,12]. One study suggested that the association between BMI-metabolic status categories and major cardiovascular events remained the same as that with BMI-insulin resistance categories [1]. Therefore, participants in our study were classified as metabolically unhealthy according to the following criteria: systolic BP ≥130 mmHg and/or diastolic BP ≥85 mmHg or treatment for hypertension, elevated TG (≥150 mg/dL), low HDL-C (men, <40 mg/dL; women, <50 mg/dL), high fasting glucose (≥100 mg/dL, with a fasting duration of at least 3 h), high casual glucose (≥140 mg/dL for less than 1 h since last meal, ≥120 mg/dL for 1 to 2 h, and ≥110 mg/dL for 2 to 3 h), or treatment for diabetes mellitus.

### Assessments of outcomes

The participants were followed from the baseline examination until the date of death or December 31, 2013. Mortality surveillance was based on the Cause-of-Death Registry at public health centers in each community with the permission of the Agency of General Affairs and the Ministry of Health, Labor, and Welfare. Information on the cause of death was coded using the International Classification of Diseases, Tenth Revision (ICD-10) codes. Cause of death was classified as follows: (1) CVD death: heart disease including sudden death (I11, I13, I34-35, I21–I23, I46, I48–I51), CVD (I60, I61, I63-64, I67, I69), and other CVD (I70-71); (2) cancer death (C03, C10, C13–C20, C22–C25, C30, C32, C34, C43-44, C48-50, C53-57, C61, C64-68, C71, C73-74, C78-80, C84-85, C90-C92, C95, D37-38, D41, D43-44, D46, D 48); and (3) other causes, such as infection and suicide.

### Statistical analysis

The participants were divided into eight phenotypes according to the BMI classification and metabolic status: metabolically healthy underweight, MUHU, metabolically healthy normal weight, MUHNW, metabolically healthy pre-obesity (MHPO), metabolically unhealthy pre-obesity (MUHPO), metabolically healthy obesity (MHO), and metabolically unhealthy obesity (MUHO). Baseline characteristics were described and compared among the eight phenotypes. Data were expressed as mean ± standard deviation (SD), except for TG as its distribution was highly skewed. TG was thus expressed as the median and interquartile range. Categorical variables, such as metabolic abnormality (increased blood pressure and impaired glucose tolerance), past history (myocardial infarction, stroke, and cancer), education attainment, married status, and smoking and drinking status, were presented as percentages. The one-way analysis of variance was used for comparison of the continuous and categorical variables between the eight phenotypes, and differences were tested via post hoc pairwise comparison with MHNW (Tukey, corresponding α value = 0.05). We examined a frequency distribution to assess the independent effects of BMI and metabolically unhealthy and used a Cox’s regression model to estimate hazard ratios (HR) and 95% confidence intervals (95% CI) for the association between the phenotypes stratified by BMI-metabolic status and mortality. A basic model (model 1) was adjusted for age, sex, total cholesterol, and smoking status (never, ex-, or current smoker), then additionally for, drinking status, education attainment (<18 years or ≥18 years), married status (yes or no), PAI, and number of hours slept (model 2). In addition to all-cause mortality, CVD and cancer-related mortality were also analyzed. We tested the proportional hazard assumption by using the log-negative-log plot of the survival function for the BMI-stratified metabolically healthy and metabolically unhealthy participants. Because inclusion of participants with existing but undiagnosed illnesses or early follow-up might bias the results, we investigated the association after exclusion of the participants who died within the first 5 years of follow-up. Using a Cox regression analysis, we separately examined whether there were different associations between younger and middle-aged participants (under 65 years) and older participants (65 years and over) among metabolically unhealthy participants. All statistical analyses were performed using SPSS version 22 (IBM, Chicago, IL, USA), and statistical significance was defined as P<0.05.

## Results

A total of 12,490 subjects (4,911 men and 7,579 women) participated in the study. From the 12,388 who completed the follow-up, 1,564 were excluded. Finally, the data from 10,824 participants were analyzed in the study. The baseline characteristics according to BMI classification and metabolic status are shown in Table 1. The study population comprised 4,261 men and 6,563 women with a mean ± SD age of 55.3±11.5 years. In total, 534, 7,720, 2,335, and 235 participants were classified as underweight, normal weight, pre-obesity, and obesity, respectively. There were 430 metabolically healthy underweight, 104 MUHU, 4,940 MHNW, 2,780 MUHNW, 896 MHPO, 1,439 MUHPO, 60 MHO, and 175 MUHO. The number of MUHNW and MUHU was higher than MUHPO and MUHO (2,884 and 1,614 participants). Among the underweight participants, 104 were identified as metabolically unhealthy (Table 1), accounting for 19.5% of the overall underweight population. The proportions of MUHNW, MUHPO, and MUHO were 36.0%, 61.6%, and 74.5%. All atherosclerosis risk factors, smoking status, and PAI were significantly different among all 8 phenotypes.

**Table 1:** Baseline clinicodemographic characteristics of the participants according to BMI/Metabolic status categories.

	All Participants	Body mass index, kg/m^2^								
		Underweight (< 18.5)		Normal weight (18.5–24.9)		Pre-obesity (25.0–29.9)		Obesity (≥30.0)		P
		Metabolically healthy	Metabolically unhealthy	Metabolically healthy	Metabolically unhealthy	Metabolically healthy	Metabolically unhealthy	Metabolically healthy	Metabolically unhealthy	
Number	10,824	430	104	4,940	2,780	896	1,439	60	175	
Age, years	55.3±11.5	55.5±15.2†	61.9±10.4‡	53.3±12.0	58.1±10.2‡	54.5±10.6	56.6±10.2‡	54.4±11.1	55.3±9.4	<0.05
Male, %	39	33	41	40	43	32‡	41	22	27*	<0.05
BMI, kg/m2	23.1±3.1	17.6±0.8‡	17.6±0.9‡	21.8±1.7	22.5±1.6‡	26.5±1.2‡	26.9±1.3‡	32.1±2.9‡	32.1±2.1‡	<0.05
Systolic BP, mmHg	129.2±20.1	115.7±18.6‡	136.8±21.4‡	120.9±18.2	138.8±18.7‡	127.3±19.4‡	141.7±19.1‡	134.0±23.3‡	143.9±19.3‡	<0.05
Diastolic BP, mmHg	77.3±12.3	69.8±10.9‡	81.1±12.9‡	72.9±11.0	81.9±11.5‡	76.9±11.3‡	84.7±11.1‡	79.6±11.7‡	86.7±11.0‡	<0.05
HDL-C, mg/dL	51.2±13.0	58.5±13.3‡	51.5±15.3*	55.5±12.0	46.4±12.6‡	53.3±10.8‡	43.1±10.6‡	53.4±9.6	43.2±11.3‡	<0.05
Triglyceride, mg/dL	96 (69, 139)	71 (58,90)†	88 (66,148)‡	81 (61,105)	127 (85,181)‡	93 (69,120)‡	157 (109,212)‡	97 (80,115) *	164 (107,230)‡	<0.05
Blood glucose, mg/dL	102.6±26.1	94.6±19.0	115.0±21.8‡	94.7±17.3	113.9±32.7‡	95.3±14.6	111.8±30.8‡	98.2±9.0	119.6±33.8‡	<0.05
Total cholesterol, mg/dL	192.2±35.0	181.3±33.5†	181.4±38.6	187.8±33.4	194.2±36.0‡	196.8±33.2‡	202.4±35.7‡	199.1±35.2	209.1±35.2‡	<0.05
Metabolic abnormalities ⁂										
Increased blood pressure, %	50	20*	79‡	27	78‡	38‡	83‡	48†	83‡	<0.05
Impaired glucose tolerance, %	31	13	72‡	11	59‡	9	55‡	15	63‡	<0.05
Past history $										
Myocardial infarction, %	1	0	2	0	1	0	1	0	1	0.209
Stroke, %	1	0	2	1	1	1	1	0	4‡	<0.05
Cancer, %	1	1	1	1	1	1	1	5	1	0.202
Smoker $										
Current, %	23	25	33	24	25	16‡	22	13	19	<0.05
EX-, %	13	11	11	12	14	13	13	8	11	0.388
Never, %	64	64	57	63	61	72‡	65	78	69	<0.05
Current drinker, % $	44	38*	39	46	43*	41*	42	30	28‡	<0.05
Sleeping hours, hours $	7.6±1.1	7.7±1.1‡	8.0±1.3‡	7.5±1.1	7.7±1.1‡	7.5±1.1	7.6±1.2‡	7.3±1.0	7.5±1.2	<0.05
Physical activity index $	33.2±7.5	32.4±6.6	32.8±7.0	33.5±7.9	33.3±7.1	32.5±6.9†	33.1±7.8	31.6±6.0	31.6±6.3*	<0.05
Education, % $	33	34	20†	37	29‡	30‡	29‡	19	24†	<0.05
Marriage, % $	92	86‡	87	92	92	93	91	95	91	<0.05

Data are expressed as mean ± standard deviation (SD), %, and median (25^th^ percentile, 75^th^ percentile). The one-way analysis of variance was used for comparison between the eight phenotypes, according to the BMI classification and metabolic status.

* P<0.05,

† P<0.01, and

‡ P<0.001 for Tukey post-hoc pairwise comparison with metabolically healthy normal weight (MHNW).

⁂Increased blood pressure: systolic BP ≥130 mmHg and/or diastolic BP ≥85 mmHg or treatment for hypertension. Impaired glucose tolerance: high fasting glucose (≥100 mg/dL), high casual glucose (≥140 mg/dL for less than 1 h since last meal, ≥120 mg/dL for 1 to 2 h, and ≥110 mg/dL for 2 to 3 h) or treatment for diabetes mellitus.

$ Data were obtained using a questionnaire.

BMI = body mass index; BP = blood pressure; HDL-C = high-density lipoprotein cholesterol.

**Fig 1:**
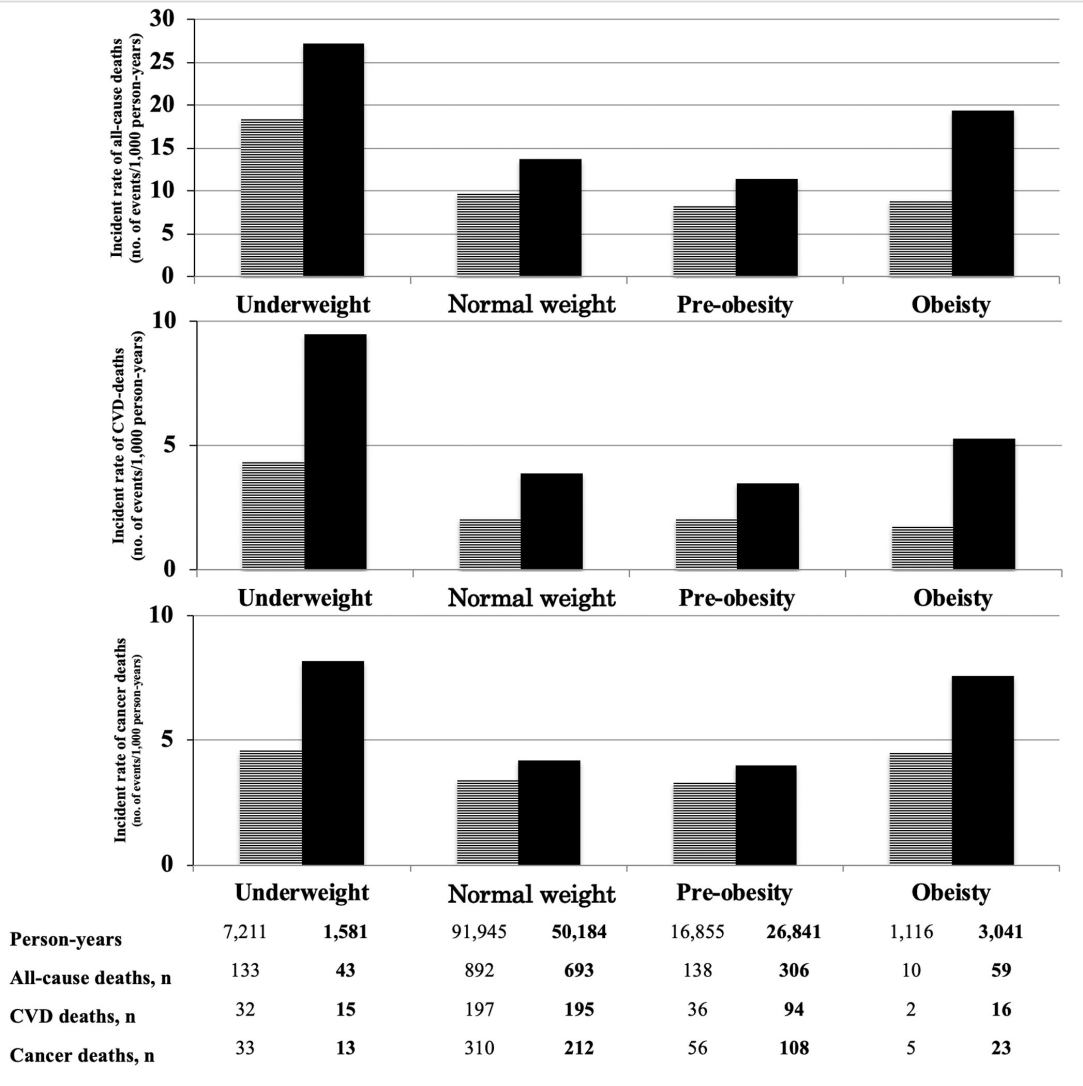
Incidence rates of all-cause deaths, cardiovascular disease (CVD) deaths, and cancer deaths in different combinations of body mass index (BMI) and metabolically unhealthy. ≡ = Metabolically healthy; ▪ = Metabolically unhealthy.

**Fig 2:**
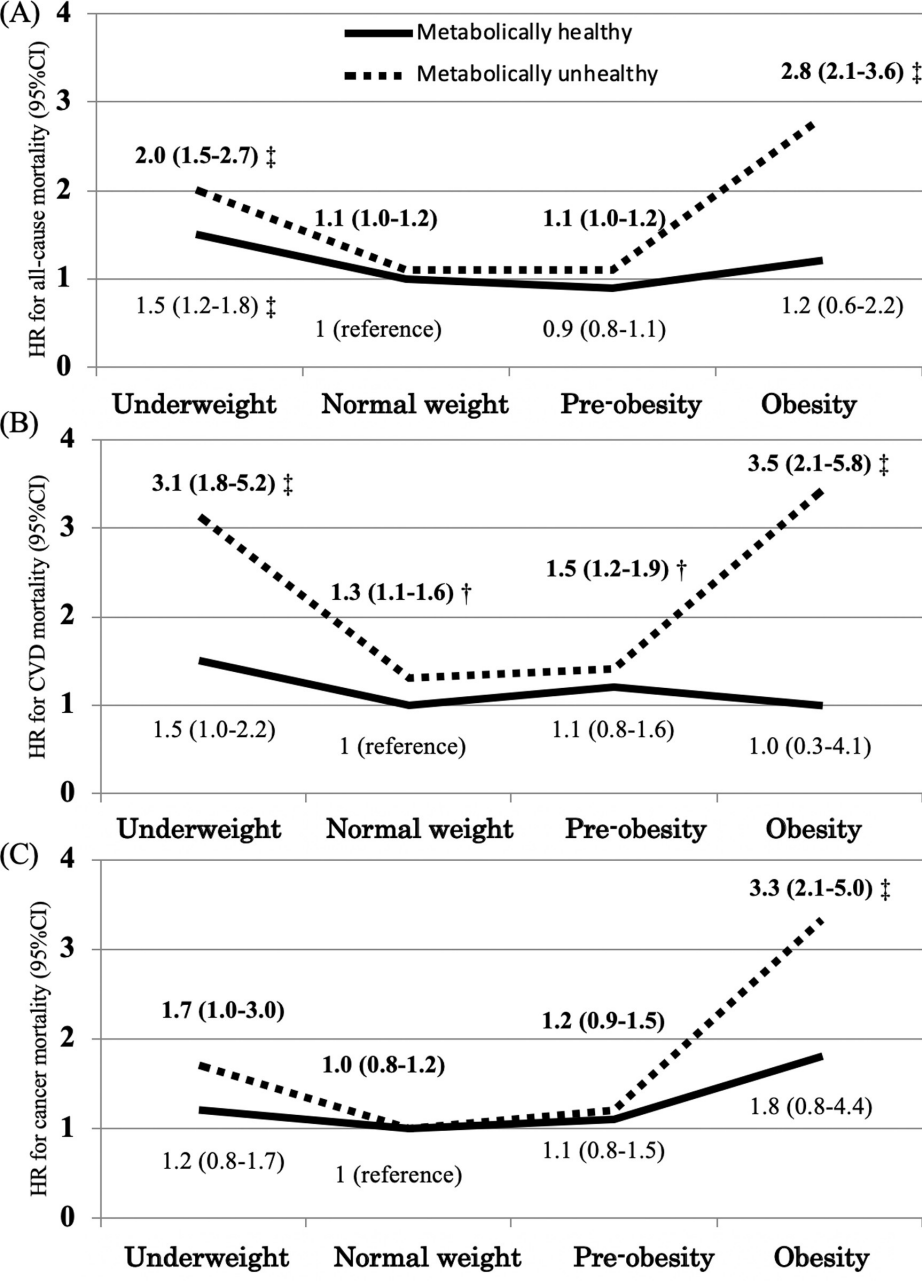
All-cause mortality (A), cardiovascular disease (CVD) mortality (B), and cancer mortality (C) in relation to body mass index (BMI) and metabolically unhealthy. Hazard ratios (HRs) and 95% confidence intervals (CIs) were calculated using a Cox regression hazard model, compared with metabolically healthy normal-weight (MHNW). Adjusted for sex, age, total cholesterol, smoking status (never, ex-, or current smoker), drinking status, education attainment (<18 years or ≥18 years), married status (yes or no), physical activity index (PAI), and sleeping hours. *P<0.05 vs. reference group, †P<0.01 vs. reference group, and ‡P<0.001 vs. reference group.

During a mean follow-up of 18.4±4.8 years (198,776 person-years), 2,274 deaths occurred (11.4 events/1,000 person-years), including 587 CVD deaths, 760 cancer deaths, and 927 other-cause deaths. Figs 1 and 2 show the person-years, events, incident rates (1,000 person-years), and HRs of deaths with a 95% CI in the different phenotypes. The incident rates of all-cause, CVD, and cancer-related death were higher among participants with underweight or obesity than those with normal weight or pre-obesity. The incident rates were also higher in metabolically unhealthy participants than in metabolically healthy participants regardless of BMI. The associations between BMI and all-cause mortality were J-shaped among metabolically unhealthy participants, but not among metabolically healthy participants, with minimum risk at normal weight and pre-obesity after adjustment for various confounders such as sex, age, total cholesterol, smoking status, drinking status, education, married status, PAI, and sleeping hours (multivariable HR [95% CI] for all-cause mortality: MUHU, 2.0 [1.5–2.7]; MUHNW, 1.1 [1.0–1.2]; MUHPO, 1.1 [1.0–1.2]; MUHO, 2.8 [2.1–3.6]) (Fig 2A). A similar pattern was seen in the prediction of cardiovascular mortality and cancer mortality (Fig 2B and 2C). The association remained the same in both sexes (S1 and S2 Figs). The results remained after excluding 278 participants with a pre-existing myocardial infarction, stroke, or cancer at baseline. Further, the results were compatible even after excluding those who died within first 5 years of follow-up (S3 Fig).

**Fig 3:**
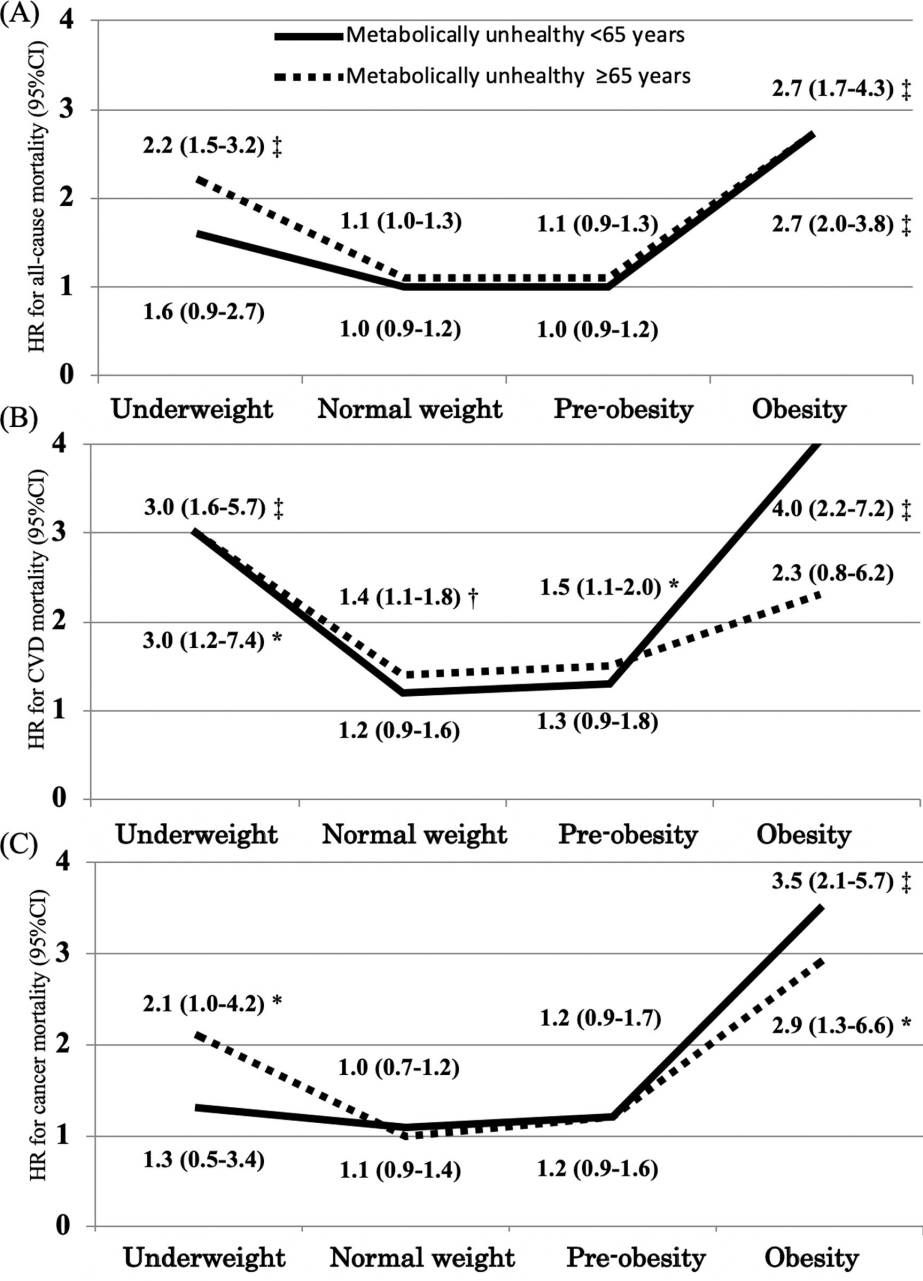
All-cause mortality (A), cardiovascular disease (CVD)-mortality (B), and cancer mortality (C) in relation to age and different combinations of body mass index (BMI) and metabolically unhealthy. Among the younger and middle-aged metabolically unhealthy participants (aged under 65 years) and older metabolically unhealthy participants (aged 65 years and over), hazard ratios (HRs) and 95% confidence intervals (CIs) were calculated using a Cox regression hazard model, compared with metabolically healthy normal weight (MHNW) in all participants. Adjusted for sex, age, total cholesterol, smoking status (never, ex-, or current smoker), drinking status, education attainment (<18 years or ≥18 years), married status (yes or no), physical activity index (PAI), and sleeping hours. *P<0.05 vs. reference group, †P<0.01 vs. reference group, and ‡P<0.001 vs. reference group.

We also separately tested whether there were different associations between the 3,271 younger and middle-aged metabolically unhealthy participants (aged under 65 years) and 1,227 older metabolically unhealthy participants (aged 65 years and over) using a Cox regression analysis (Tables 2 and 3 and Fig 3). MHNW in all participants was used as the reference category. The associations remained the same regardless of age. However, among metabolically unhealthy participants aged <65 years, all-cause and cancer mortality was not significant in underweight (multivariable HR [95% CI] for all-cause mortality, 1.6 [0.9–2.7]; for cancer mortality, 1.3 [0.5–3.4]) (Table 2 and Fig 3). By contrast, among metabolically unhealthy participants aged ≥65 years, CVD mortality was not significant in obesity (multivariable HR [95% CI] for CVD-mortality, 2.3 [0.8–6.2]) (Table 3 and Fig 3).

**Table 2:** All-Cause Mortality, Cardiovascular Disease (CVD) Mortality, and Cancer Mortality in Relation to Body Mass Index (BMI) and Metabolically Unhealthy in Participants Aged <65 Years (N = 3,271).

	Body mass index, kg/m^2^							
	Underweight (< 18.5)		Normal weight (18.5–24.9)		Pre-obesity (25.0–29.9)		Obesity (≥30.0)	
	Metabolically healthy	Metabolically unhealthy	Metabolically healthy	Metabolically unhealthy	Metabolically healthy	Metabolically unhealthy	Metabolically healthy	Metabolically unhealthy
Number	281	**59**	4,940	**1,971**	720	**1,096**	50	**145**
**All-cause death**								
Person-years	5,120	**1,018**	91,945	**37,034**	13,869	**20,959**	950	**2,595**
Number of cases	42	**14**	892	**314**	75	**161**	6	**39**
Incidence rate ^a^	8.2	**13.8**	9.7	**8.5**	5.4	**7.7**	6.3	**15.0**
HR (95%CI), model 1	1.3 (1.0–1.8)	**1.5 (0.9–2.5)**	1 (reference)	**1.0 (0.9–1.2)**	0.9 (0.7–1.1)	**1.1 (0.9–1.3)**	1.2 (0.5–2.6)	**2.6 (1.9–3.7)** **‡**
HR (95%CI), model 2	1.3 (0.9–1.8)	**1.6 (0.9–2.7)**	1 (reference)	**1.0 (0.9–1.2)**	0.9 (0.7–1.1)	**1.0 (0.9–1.2)**	1.3 (0.6–2.9)	**2.7 (2.0–3.8)** **‡**
**CVD death**								
Number of cases	9	**5**	197	**74**	16	**41**	1	**12**
Incidence rate ^a^	1.8	**4.9**	2.1	**2.0**	1.2	**2.0**	1.1	**4.6**
HR (95%CI), model 1	1.5 (0.8–3.0)	**2.7 (1.1–6.6)** *	1 (reference)	**1.2 (0.9–1.6)**	0.9 (0.6–1.6)	**1.3 (0.9–1.9)**	1.0 (0.1–7.0)	**4.0 (2.2–7.3)** **‡**
HR (95%CI), model 2	1.4 (0.7–3.0)	**3.0 (1.2–7.4)** *	1 (reference)	**1.2 (0.9–1.6)**	1.0 (0.6–1.6)	**1.3 (0.9–1.8)**	1.0 (0.1–7.2)	**4.0 (2.2–7.2)** **‡**
**Cancer death**								
Number of cases	14	**5**	310	**122**	37	**62**	3	**17**
Incidence rate ^a^	2.7	**4.9**	3.4	**3.3**	2.7	**3.0**	3.2	**6.6**
HR (95%CI), model 1	1.1 (0.7–1.9)	**1.4 (0.6–3.5)**	1 (reference)	**1.1 (0.9–1.4)**	1.2 (0.9–1.7)	**1.1 (0.9–1.5)**	1.6 (0.5–5.1)	**3.3 (2.0–5.3)** **‡**
HR (95%CI), model 2	1.1 (0.6–2.0)	**1.3 (0.5–3.4)**	1 (reference)	**1.1 (0.9–1.4)**	1.2 (0.8–1.7)	**1.2 (0.9–1.6)**	1.8 (0.6–5.6)	**3.5 (2.1–5.7)** **‡**

Hazard ratios (HRs) and 95% confidence intervals (CIs) were calculated using a Cox regression hazard model, compared with metabolically healthy normal weight (MHNW) in all participants. Adjusted for sex, age, total cholesterol, and smoking status (never, ex-, or current smoker) (model 1) and additionally for drinking status, education attainment (<18 years or ≥18 years), married status (yes or no), physical activity index (PAI), and sleeping hours (model 2).

*P<0.05 vs. reference group,

†P<0.01 vs. reference group,

‡P<0.001 vs. reference group.

a: Per 1,000 person-years.

**Table 3:** All-Cause Mortality, Cardiovascular Disease (CVD)-Mortality, and Cancer Mortality in Relation to Body Mass Index (BMI) and Metabolically Unhealthy in Participants Aged ≥65 Years (N = 1,227).

	Body mass index, kg/m^2^							
	Underweight (< 18.5)		Normal weight (18.5–24.9)		Pre-obesity (25.0–29.9)		Obesity (≥30.0)	
	Metabolically healthy	Metabolically unhealthy	Metabolically healthy	Metabolically unhealthy	Metabolically healthy	Metabolically unhealthy	Metabolically healthy	Metabolically unhealthy
Number	149	**45**	4,940	**809**	176	**343**	10	**30**
**All-cause death**								
Person-years	2,092	**563**	91,945	**13,150**	2,986	**5,882**	166	**446**
Number of cases	91	**29**	892	**379**	63	**145**	4	**20**
Incidence rate ^a^	43.5	**51.5**	9.7	**28.8**	21.1	**24.7**	24.1	**44.8**
HR (95%CI), model 1	1.6 (1.2–1.9) ‡	**2.1 (1.4–3.0)** ‡	1 (reference)	**1.1 (1.0–1.2)**	1.0 (0.7–1.3)	**1.1 (0.9–1.3)**	0.9 (0.3–2.4)	**2.9 (1.9–4.6)** ‡
HR (95%CI), model 2	1.6 (1.2–2.0) ‡	**2.2 (1.5–3.2)** ‡	1 (reference)	**1.1 (1.0–1.3)**	1.0 (0.8–1.3)	**1.1 (0.9–1.3)**	1.0 (0.4–2.7)	**2.7 (1.7–4.3)** ‡
**CVD death**								
Number of cases	23	**10**	197	**121**	20	**53**	1	**4**
Incidence rate ^a^	11.0	**17.8**	2.1	**9.2**	6.7	**9.0**	6.0	**9.0**
HR (95%CI), model 1	1.5 (1.0–2.3)	**2.8 (1.5–5.4)** **†**	1 (reference)	**1.3 (1.1–1.7)** *	1.2 (0.8–1.9)	**1.5 (1.1–2.1)** **†**	0.8 (0.1–6.0)	**2.4 (0.9–6.4)**
HR (95%CI), model 2	1.4 (0.9–2.3)	**3.0 (1.6–5.7)** ‡	1 (reference)	**1.4 (1.1–1.8)** **†**	1.3 (0.8–2.0)	**1.5 (1.1–2.0)** *	0.9 (0.1–6.5)	**2.3 (0.8–6.2)**
**Cancer death**								
Number of cases	19	**8**	310	**90**	19	**46**	2	**6**
Incidence rate ^a^	9.1	**14.2**	3.4	**6.8**	6.4	**7.8**	12.0	**13.5**
HR (95%CI), model 1	1.2 (0.7–1.9)	**1.9 (0.9–3.9)**	1 (reference)	**0.9 (0.7–1.2)**	1.0 (0.7–1.2)	**1.3 (0.9–1.7)**	1.9 (0.5–7.6)	**2.9 (1.3–6.7)** **†**
HR (95%CI), model 2	1.2 (0.8–2.0)	**2.1 (1.0–4.2)** *	1 (reference)	**1.0 (0.7–1.2)**	1.0 (0.6–1.6)	**1.2 (0.9–1.7)**	2.2 (0.5–9.0)	**2.9 (1.3–6.6)** *

Hazard ratios (HRs) and 95% confidence intervals (CIs) were calculated using a Cox regression hazard model, compared with metabolically healthy normal weight (MHNW) in all participants. Adjusted for sex, age, total cholesterol, and smoking status (never, ex-, or current smoker) (model 1) and additionally for drinking status, education attainment (<18 years or ≥18 years), married status (yes or no), physical activity index (PAI), and sleeping hours (model 2).

*P<0.05 vs. reference group,

†P<0.01 vs. reference group,

‡P<0.001 vs. reference group.

a: Per 1,000 person-years.

## Discussion

This study investigated the associations of BMI and metabolically unhealthy weight with all-cause mortality, CVD mortality, and cancer mortality. The results showed that BMI and metabolically unhealthy is associated with mortality in a rural Japanese general population. To the best of our knowledge, our study is the first to demonstrate such association. Specifically, the main findings were that (1) MUHNW and MUHU were not rare in Japan. MUHNW and MUHU had higher BP, blood glucose, and lipid levels than MHO, although they probably had lower fat mass. (2) MUHNW and MUHU had a 1.3- to 3.1-fold higher risk of CVD mortality. MUHU was more strongly associated with an increased risk of CVD mortality, and the association remained the same regardless of age. (3) Among metabolically unhealthy participants, the associations between BMI and all-cause mortality, CVD mortality, and cancer mortality are J-shaped, regardless of sex and age. Exceptionally, an inverted J-shaped relationship was noted between BMI and CVD mortality was among metabolically unhealthy participants aged ≥65 years. These results indicate the necessity for a more careful evaluation of not only MUHO but also MUHU, regardless of sex and age.

Previous studies reported increased risks of respiratory-, mental disorder related-, musculoskeletal disease related-, injury-related, external cause-related, and cancer-related mortalities in the underweight population [27]. Such findings were attributed to poor self-care, low economic status, and mental health problems. In our study, metabolically unhealthy was also not rare among the underweight population, and this contributed to the increased risk of CVD mortality. Therefore, we should also pay attention to the underlying mechanisms of insulin resistance and metabolic dysfunction among subjects with normal weight and underweight to provide individualized preventive care and treatment based on the disease pathophysiology. Although the mechanisms for the association of BMI and metabolically unhealthy with mortality have not been clearly elucidated, several potential pathways may explain this association. A lipodystrophy-like phenotype, which is significantly associated with abnormalities in lipid storage (e.g., lower leg fat mass, impaired insulin secretion capacity, and insulin resistance) and decreased cardiorespiratory fitness, may exist in the general population [12]. The analyses of the genetic and molecular mechanisms of insulin resistance showed that the mechanism to being metabolically unhealthy might differ between those with and without obesity [28,29]. This may result in the impaired distribution of adipose tissue and promotion of adipocyte differentiation, which leads to the increased risk of CVD and mortality [12]. Other possible explanations for the underlying mechanisms would include decreased protective effect by skeletal muscle loss, altered hormonal milieu and autonomic nerve system function, and increased systemic inflammation [30,31,32].

Although our results could not identify the exact mechanism, we found that physical activity factors such as PAI and sleep time might not be related to the different mechanisms of insulin resistance and metabolic dysfunction among subjects with/without obesity. The metabolically unhealthy subjects had comparable PAI and sleep time with metabolically healthy subjects.

We could not also determine the reason by which the risk of all-cause, CVD, and cancer mortality was higher, regardless of age, in the MUHU than in the MUHNW. It might be possible that the mechanisms mentioned above, that is, a catabolic/anabolic imbalance and a vicious cycle worsening the nutritional status, could be the cause. There might also be survival bias. Smoking is associated with lower body weight and a strong risk factor for CVD and cancer-mortality [33,34]. We assessed the associations after excluding current and ex-smokers. The results were compatible, although the assessment of the results was limited because majority of participants in the analysis were women (S4 Fig).

The strengths of our study include the following: 1) its population-based design focused on the Japanese population, which addresses the limited available evidence on the risk of mortality among BMI-stratified metabolically unhealthy subjects in the Asian population; 2) almost all participants completed the follow-up; and 3) a long-term follow-up period (average: 18.4±4.8 years).

However, our study also has several limitations that should be considered when interpreting the findings. First, because there is no consensus on the definition of metabolically unhealthy, there might be heterogeneity among metabolically unhealthy participants. However, as detailed in the Methods section, we classified the metabolically unhealthy according to the criterion used by most studies, most of which have relatively low heterogeneity. Second, we could not rule out the possibility that metabolically healthy participants at baseline developed metabolic dysfunctions during follow-up, which led to the increased mortality. We could not evaluate the effects of change in body weight and other factors during follow-up because data on BMI and other atherosclerosis factors were available only at baseline. Third, the criteria used to define high casual glucose (i.e., ≥140 mg/dL for less than 1 h since last meal, ≥120 mg/dL for 1 to 2 h, and ≥110 mg/dL for 2 to 3 h) makes it difficult to assess the association between casual glucose and impaired glucose tolerance because casual glucose is not markedly affected by the severity of impaired glucose tolerance classified by fasting glucose or 75-g oral glucose tolerance. In the present study, we used the casual glucose levels, which are equal to a fasting glucose of 100 mg/dL, calculated using the distribution data of the casual glucose and daily glucose profiles in subjects with normal glucose tolerance. Fourth, it is very difficult whether we use “elevated TG (≥150 mg/dL)” to define MetS in non-fasting state, because there have been no standardized references for postprandial TG levels to define MetS in non-fasting state. A population study suggested that the prevalence of MetS calculated by using the postprandial TG values instead of the fasting TG values was almost as same as the prevalence by using the fasting TG [35]. Therefore, in our study, we used “elevated TG (≥150 mg/dL), low HDL-C (men, <40 mg/dL; women, <50 mg/dL)” to define MetS in non-fasting state. However, it might be possible that the MetS misclassification by using the postprandial TG values instead of the fasting TG values led to the underestimate of the risk of death in our study. So, using the reference for elevated TG to define the fasting hypertriglyceridemia and the postprandial hypertriglyceridemia (≥150 mg/dL, with a fasting duration of at least 6 h, and ≥175 mg/dL, for 1 to 6 hours since last meal), the prevalence of MetS was 32.5% (using the reference for elevated TG (≥150 mg/dL, in fasting and non-fasting state); 34.0%), and the main result remained the same (S5 Fig). The postprandial hypertriglyceridemia was proposed by the European Atherosclerosis Society and European Federation of Clinical Chemistry and Laboratory Medicine [36]. Finally, the assessment of the results of subgroup analysis is limited because of smaller events in the subgroups.

## Conclusions

Our study showed that metabolically unhealthy was not rare among the underweight and normal weight population in Japan. Further, not only MUHNW but also MUHU are associated with an increased risk of CVD mortality. Among metabolically unhealthy individuals, the association between BMI and all-cause mortality is J-shaped regardless of age and sex. Therefore, the health issues of not only MUHO, but also MUHU should be given attention. The association and underlying mechanism of insulin resistance and metabolic dysfunction in the underweight population is yet to be identifies, and thus it is difficult to provide pathophysiology-based individualized preventive care and treatment for metabolic dysfunction. Further studies are necessary to understand the pathophysiology more precisely and achieve an individualized approach to treatment.

## Supporting information

S1 Fig **All-cause mortality (A), cardiovascular disease (CVD)-mortality (B), and cancer mortality (C) in relation to age and different combinations of body mass index (BMI) and metabolically unhealthy in men.** Hazard ratios (HRs) and 95% confidence intervals (CIs) were calculated using a Cox regression hazard model, compared with metabolically healthy normal-weight (MHNW). Adjusted for sex, age, total cholesterol, smoking status (never, ex-, or current smoker), drinking status, education attainment (<18 years or ≥18 years), married status (yes or no), physical activity index (PAI), and sleeping hours. *P<0.05 vs. reference group, †P<0.01 vs. reference group, and ‡P<0.001 vs. reference group.
(PDF)

S2 Fig **All-cause mortality (A), cardiovascular disease (CVD)-mortality (B), and cancer mortality (C) in relation to age and different combinations of body mass index (BMI) and metabolically unhealthy in women.** Hazard ratios (HRs) and 95% confidence intervals (CIs) were calculated using a Cox regression hazard model, compared with metabolically healthy normal-weight (MHNW). Adjusted for sex, age, total cholesterol, smoking status (never, ex-, or current smoker), drinking status, education attainment (<18 years or ≥18 years), married status (yes or no), physical activity index (PAI), and sleeping hours. *P<0.05 vs. reference group, †P<0.01 vs. reference group, and ‡P<0.001 vs. reference group.
(PDF)

S3 Fig **All-cause mortality (A), cardiovascular disease (CVD) mortality (B), and cancer mortality (C) in relation to body mass index (BMI) and metabolically unhealthy after excluding participants with a pre-existing myocardial infarction, stroke, or cancer at baseline and/or who died within first 5 years of follow-up.** Hazard ratios (HRs) and 95% confidence intervals (CIs) were calculated using a Cox regression hazard model, compared with metabolically healthy normal-weight (MHNW). Adjusted for sex, age, total cholesterol, smoking status (never, ex-, or current smoker), drinking status, education attainment (<18 years or ≥18 years), married status (yes or no), physical activity index (PAI), and sleeping hours. *P<0.05 vs. reference group, †P<0.01 vs. reference group, and ‡P<0.001 vs. reference group.
(PDF)

S4 Fig **All-cause mortality (A), cardiovascular disease (CVD) mortality (B), and cancer mortality (C) in relation to body mass index (BMI) and metabolically unhealthy after excluding current and ex-smokers.** Hazard ratios (HRs) and 95% confidence intervals (CIs) were calculated using a Cox regression hazard model, compared with metabolically healthy normal-weight (MHNW). Adjusted for sex, age, total cholesterol, smoking status (never, ex-, or current smoker), drinking status, education attainment (<18 years or ≥18 years), married status (yes or no), physical activity index (PAI), and sleeping hours. *P<0.05 vs. reference group, †P<0.01 vs. reference group, and ‡P<0.001 vs. reference group.
(PDF)

S5 Fig **All-cause mortality (A), cardiovascular disease (CVD) mortality (B), and cancer mortality (C) in relation to body mass index (BMI) and metabolically unhealthy using the reference for elevated triglyceride to define the postprandial hypertriglyceridemia.** Hazard ratios (HRs) and 95% confidence intervals (CIs) were calculated using a Cox regression hazard model, compared with metabolically healthy normal-weight (MHNW). Adjusted for sex, age, total cholesterol, smoking status (never, ex-, or current smoker), drinking status, education attainment (<18 years or ≥18 years), married status (yes or no), physical activity index (PAI), and sleeping hours. *P<0.05 vs. reference group, †P<0.01 vs. reference group, and ‡P<0.001 vs. reference group.
(PDF)
