# Supplementary material for: Impact of body mass index and metabolically unhealthy status on mortality in the Japanese general population: The JMS cohort study
Source: PLoS One. 2019 Nov 7;14(11):e0224802. doi: 10.1371/journal.pone.0224802 (PMC6837339; doi:10.1371/journal.pone.0224802)
Supplement: S1 Fig — All-cause mortality (A), cardiovascular disease (CVD)-mortality (B), and cancer mortality (C) in relation to age and different combinations of body mass index (BMI) and metabolically unhealthy in men. Hazard ratios (HRs) and 95% confidence intervals (CIs) were calculated using a Cox regression hazard model, compared with metabolically healthy normal-weight (MHNW). Adjusted for sex, age, total cholesterol, smoking status (never, ex-, or current smoker), drinking status, education attainment (<18 years or ≥18 years), married status (yes or no), physical activity index (PAI), and sleeping hours. *P<0.05 vs. reference group, †P<0.01 vs. reference group, and ‡P<0.001 vs. reference group. (PDF) [file pone.0224802.s001.pdf]

S Fig 1. All-cause mortality (A), cardiovascular disease (CVD)-mortality (B), and cancer mortality (C) in relation to age and different combinations of body mass index (BMI) and metabolically unhealthy in men.

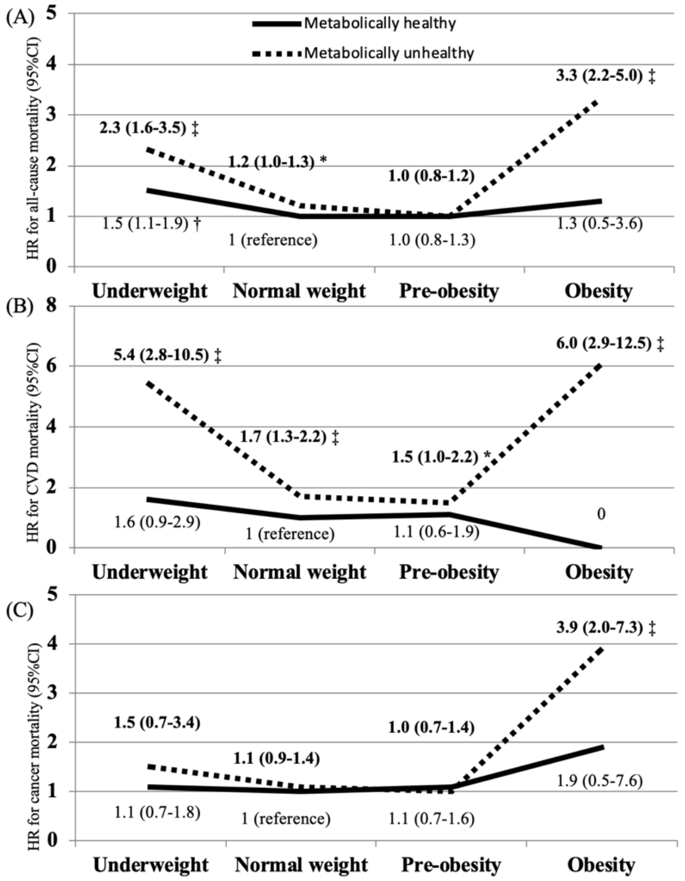

Hazard ratios (HRs) and 95% confidence intervals (CIs) were calculated using a Cox regression hazard model, compared with metabolically healthy normal-weight (MHNW). Adjusted for sex, age, total cholesterol, smoking status (never, ex-, or current smoker), drinking status, education attainment (<18 years or ≥18 years), married status (yes or no), physical activity index (PAI), and sleeping hours. *P<0.05 vs. reference group, †P<0.01 vs. reference group, and ‡P<0.001 vs. reference group.
