# Supplementary material for: Impact of body mass index and metabolically unhealthy status on mortality in the Japanese general population: The JMS cohort study
Source: PLoS One. 2019 Nov 7;14(11):e0224802. doi: 10.1371/journal.pone.0224802 (PMC6837339; doi:10.1371/journal.pone.0224802)
Supplement: S5 Fig — All-cause mortality (A), cardiovascular disease (CVD) mortality (B), and cancer mortality (C) in relation to body mass index (BMI) and metabolically unhealthy using the reference for elevated triglyceride to define the postprandial hypertriglyceridemia. Hazard ratios (HRs) and 95% confidence intervals (CIs) were calculated using a Cox regression hazard model, compared with metabolically healthy normal-weight (MHNW). Adjusted for sex, age, total cholesterol, smoking status (never, ex-, or current smoker), drinking status, education attainment (<18 years or ≥18 years), married status (yes or no), physical activity index (PAI), and sleeping hours. *P<0.05 vs. reference group, †P<0.01 vs. reference group, and ‡P<0.001 vs. reference group. (PDF) [file pone.0224802.s005.pdf]

S Fig 5. All-cause mortality (A), cardiovascular disease (CVD) mortality (B), and cancer mortality (C) in relation to body mass index (BMI) and metabolically unhealthy using the reference for elevated triglyceride to define the postprandial hypertriglyceridemia.

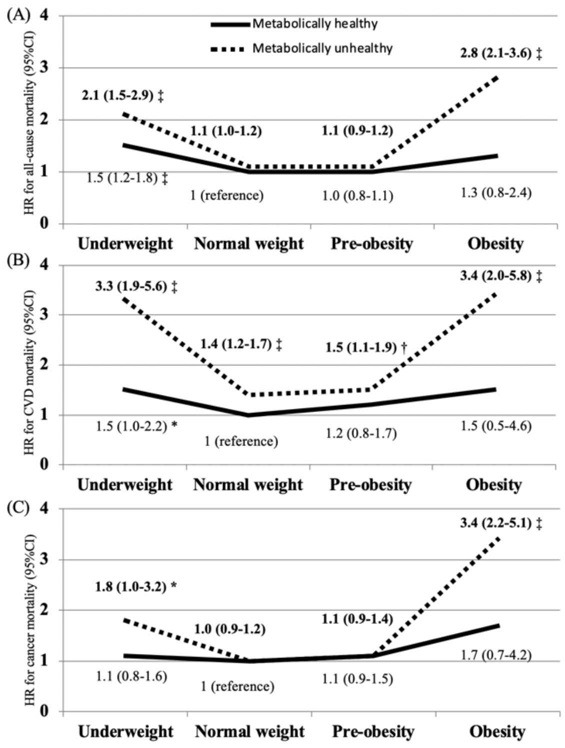

Hazard ratios (HRs) and 95% confidence intervals (CIs) were calculated using a Cox regression hazard model, compared with metabolically healthy normal-weight (MHNW). Adjusted for sex, age, total cholesterol, smoking status (never, ex-, or current smoker), drinking status, education attainment (<18 years or ≥18 years), married status (yes or no), physical activity index (PAI), and sleeping hours. *P<0.05 vs. reference group, †P<0.01 vs. reference group, and ‡P<0.001 vs. reference group.
